# The sub-fossil diatom distribution in the Beibu Gulf (northwest South China Sea) and related environmental interpretation

**DOI:** 10.7717/peerj.13115

**Published:** 2022-05-16

**Authors:** Jinpeng Zhang, Andrzej Witkowski, Michał Tomczak, Chao Li, Kevin McCartney, Zhen Xia

**Affiliations:** 1 Guangzhou Marine Geological Survey, China Geological Survey/ Southern Marine Science and Engineering Guangdong Laboratory (Guangzhou), Guangzhou, China; 2 Institute of Marine and Environmental Sciences, University of Szczecin, Szczecin, Poland; 3 Polish Geological Institute, National Research Institute, Warsaw, Poland; 4 College of Ocean and Earth Sciences, Xiamen University, Xiamen, China; 5 Department of Environmental Science and Sustainability, University of Maine at Presque Isle, Presque Isle, Maine, USA

**Keywords:** Diatoms, South China Sea, Oceanographic variables, Paleoceanography, Paleoenvironmental proxies, Multivariate analyses

## Abstract

Located in northwestern South China Sea (SCS), the Beibu Gulf constitutes an environmentally sensitive region shaped by land-ocean-atmosphere interactions in Asia between the western Pacific and eastern Indian Oceans. This study aims to provide a comprehensive view of the sub-fossil diatom biogeography, distribution pattern and oceanographic environmental controls with support of multivariate methods based on Beibu Gulf core-top samples. Cluster analysis of diatom assemblages divides the distribution pattern into four subclusters. Sea surface salinity (SSS), temperature (SST), trophic state (chlorophyll *a* concentration in this study) and water depth constrain the diatom distribution pattern through canonical redundancy analysis although only partly support an interpretation of the relationship between these various variables. Chlorophyll *a* has a strong correlation to diatom distribution, and responds to *Paralia sulcata* occurrence, while SSS and SST also have significant influence and indicate warm water invasion from the open SCS. Water depth is a subordinate factor in terms of Beibu Gulf diatom distribution. The ca. 25 m water-depth marks the upper extent of *Paralia sulcata* dominance in the northern Beibu Gulf. A strong mixing area with a complex diatom distribution exists below this water depth in the middle of Beibu Gulf. Coastal currents from north of SCS invade Beibu Gulf through Qiongzhou Strait and south of Hainan Island, as recorded by higher percentages of *Paralia sulcata* and *Cyclotella striata* at these sites. Our results provide a selection of evaluation method for a marine ecological red-line definition for sustainable development. This study highlights the perspective relationships between the spatial distribution of sub-fossil diatom assemblages in surface sediments and oceanographic variables, which could serve as a model for paleoenvironmental and paleoclimatic reconstruction in future marginal sea geoscience research for the Beibu Gulf, northwestern SCS.

## Introduction

**Figure 1:**
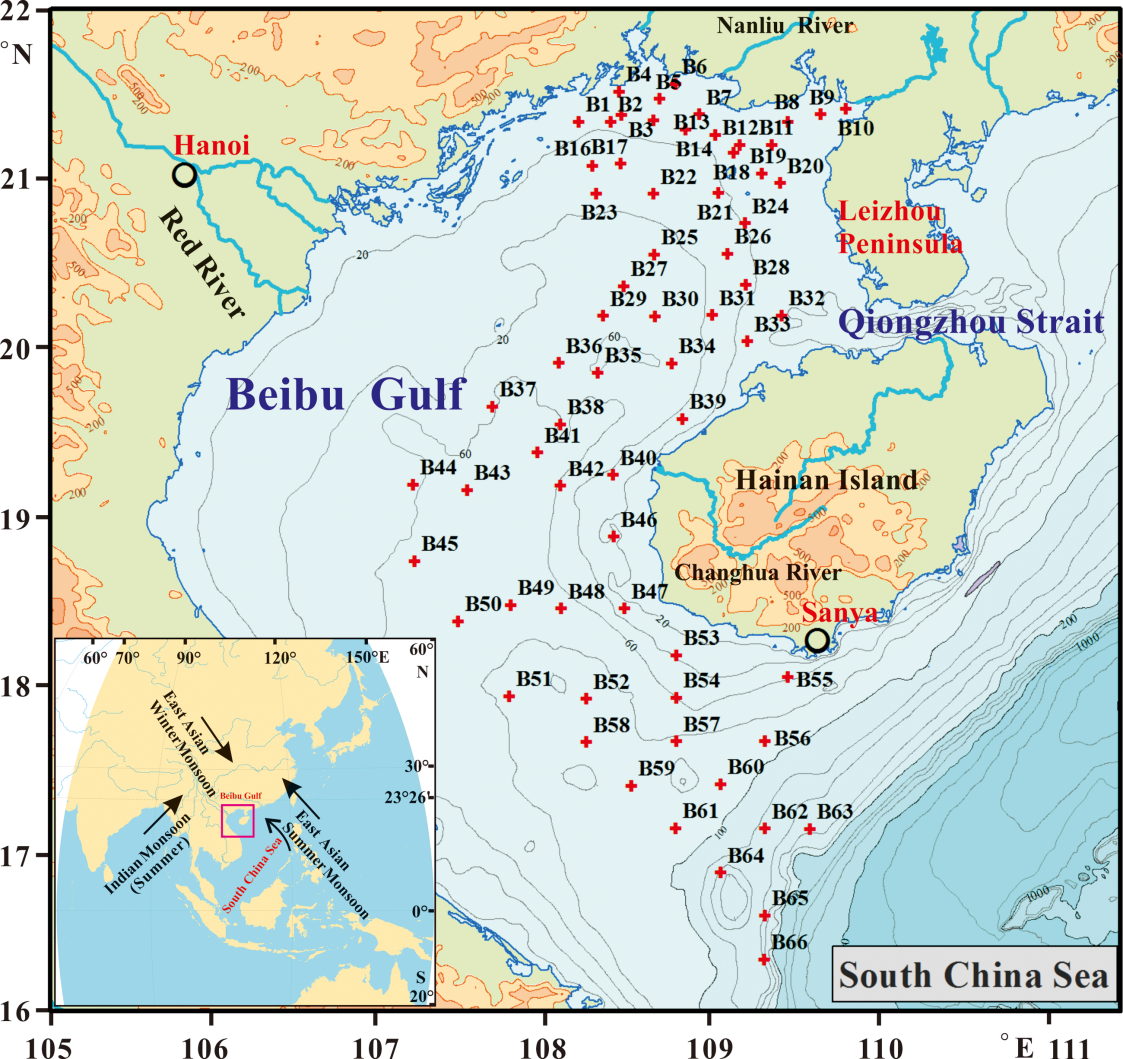
Location of sampling sites in the Beibu Gulf.

Research on sub-fossil diatom distribution in the continental marginal seas has received increased attention from the scientific community, which seeks to understand past oceanographic environments and sea-land interaction. These studies show the potential for environmental reconstruction of the relationships between diatoms and oceanographic variables such as salinity (Jiang, 1996; Zong et al., 2010), temperature (Esper, Gersonde & Kadagies, 2010; Świło et al., 2016), eutrophication (Gronlund, 1993; Mazzei & Gaiser, 2018), sea ice cover ratio (Sha et al., 2014; Krawczyk et al., 2017), currents (Bao, Manuel & Ricardo, 1997; Cárdenas et al., 2019), and sea level change (Witkowski et al., 2005; Szkornik, Gehrels & Kirby, 2006). As a large Eastern Asian gulf in the subtropical to tropical transition zone of the South China Sea (SCS), the Beibu Gulf receives river discharge from mainland China, Indo-China Peninsula and Hainan Island. Besides the largest Red River runoff from the west, other rivers provide runoff over shorter distances around this gulf. The Beibu Gulf water circulation is characterized by sea-water exchange from two directions: (1) Qiongzhou Strait in northeastern Beibu Gulf, and (2) a transitional zone in the south directly linked with SCS (Fig. 1).

The complex oceanographic conditions in this half-closed shelf water region result in a diversity of phytoplankton assemblages. Zhou et al. (2008) and Zhou, Chen & Gao (2009) reported that diatom taxa constitute ca. 74% of Beibu Gulf phytoplankton in the water column. Presently, the distribution of sub-fossil diatom assemblages in Beibu Gulf surface sediments is poorly known and at some places unknown. Jiang (1987) published the diatom composition in 10 surface sediment samples in northeast Beibu Gulf, with large quantity of marine-brackish water diatom species (*e.g.*, *Cyclotella striata* and *Paralia sulcata*; full names listed in Table 1). Chen et al. (2011) studied diatoms from a small river (Beilun River) mouth along the inland course. Zhang, Chen & Zhang (2013) studied the diatom distribution on the seafloor in northwest SCS, with samples that include a few sites in proximity of Beibu Gulf and showed occurrence of tropical species (*e.g.*, *Azpeitia nodulifera*) with diatom species from shallow waters characterized by a gradient corresponding to environmental variables.

**Table 1:** Dominant diatom species with relative abundances and their preferred habitat (based on Jin, Cheng & Lin, 1982; Jin, Cheng & Liu, 1992; Lan, Chen & Liu, 1995; Witkowski, Lange-Bertalot & Metzeltin, 2000; Guo & Qian, 2003; Winter, 2001).

Species	Temperature	Salinity	Relative abundances	Average value
**Dominant:**				
*Actinocyclus octonarius* (Ehrenberg) Kützing, 1844	wide	marina-neritic	0–8.22%	3.47%
*Actinoptychus senarius* (Ehrenberg) Ehrenberg, 1843	wide	marina-neritic	0–6.16%	1.21%
*Azpeitia nodulifera* (Janisch) G. Fryxell & T.P. Watkins, 1986	tropical	marina-open sea	0.54–13.24%	4.03%
*Campylodiscus brightwellii* Stodder, 1861	warm	marina-neritic	0–21.45%	5.94%
*Coscinodiscus argus* Ehrenberg, 1839	wide	marina-neritic	0–10.03%	2.53%
*Cyclotella striata* (Kützing) Grunow, 1880	wide	brackish-coastal	3.46–46.06%	21.17%
*Cyclotella stylorum* Brightwell, 1860	wide	brackish-coastal	0.81–21.21%	8.11%
*Diploneis bombus* (Ehrenberg) Ehrenberg, 1853	wide	brackish-coastal	0–5.71%	0.78%
*Diploneis crabro* (Ehrenberg) Ehrenberg, 1854	wide	brackish-coastal	0–8.13%	1.18%
*Hyalodiscus radiatus* (O’Meara) Grunow, 1880	wide	marina-neritic	0–7.78%	0.62%
*Paralia sulcata* (Ehrenberg) Cleve, 1873	wide	brackish-coastal	3.95–54.8%	17.97%
*Planktoniella blanda* (A. Schmidt) Syvertsen & Hasle, 1993	warm	marina-neritic	0–14.78%	3.57%
*Pleurosigma* spp*.*	wide	brackish-coastal	0–8.47%	1.89%
*Podosira stelligera* (Bailey) A.Mann, 1907	wide	marina-neritic	0–8.82%	2.20%
*Stephanopyxis weyprechtii* (Grunow) M. Hajós	warm	marina-neritic	0–10.29%	1.92%
*Surirella fluminensis* Grunow, 1862	wide	marina-neritic	0–8.29%	1.46%
*Thalassiosira bipartita* (Rattray) Hallegraeff, 1992	warm	marina-neritic	0–6.02%	1.41%
*Trachyneis antillarum* (Cleve & Grunow) Cleve, 1894	wide	marina-neritic	0–5.0%	1.41%
*Triceratium favus* Ehrenberg, 1839	wide	marina-neritic	0.28–10.71%	2.09%
*Triceratium reticulum * Ehrenberg, 1844	wide	marina-neritic	0–5.77%	0.32%
**Less abundant (also listed in text):**				
*Asterolampra vanheurckii* Brun, 1891	warm	marina-neritic	0–0.13%	0.002%
*Asteromphalus flabellatus* (Brébisson) Greville, 1859	warm	marina	0–0.81%	0.16%
*Azpeitia africana* (Janisch ex A.Schmidt) G.Fryxell & T.P.Watkins, 1986	tropical	marina-open sea	0–0.69%	0.06%
*Azpeitia neocrenulata* (Van Landingham) Fryxell & T.P.Watkins, 1986	tropical	marina-open sea	0–0.29%	0.05%
*Bacteriastrum hyalinum* Lauder, 1864	warm	marina-neritic	0-3.18%	0.28%
*Cymbella* sp.	wide	fresh water	0–0.24%	0.004%
*Ditylum brightwellii* (T. West) Grunow, 1885	warm	marina-neritic		
*Hemidiscus cuneiformis* Wallich, 1860	tropical	marina-open sea	0–4.86%	1.17%
*Planothidium hauckianum* (Grunow) Bukhtiyarova, 1996	wide	fresh water	0–0.26%	0.004%
*Rhizosolenia bergonii* H. Peragallo, 1892	tropical	marina-neritic	0–4%	0.33%
*Thalassiosira leptopus* (Grunow) Hasle & G.Fryxell, 1977	warm	marina	0–1.48%	0.24%
*Trachyneis aspera* (Ehrenberg) Cleve, 1894	wide	brackish-coastal	0–3.26%	0.75%
*Trieres chinensis* (Greville) Ashworth & E.C. Theriot, 2013	warm	marina-neritic	0–2.32%	0.27%

This article presents the distribution pattern of sub-fossil diatom assemblages in Beibu Gulf, northwest SCS supported by multivariate analyses, as Q-mode and R-mode hierarchical analyses, principal component analysis, correspondence analysis and redundancy analysis as well. Special attention is paid to eastern Beibu Gulf that surround the western and southern coasts of Hainan Island. Presented are absolute and relative diatom species abundances and spatial relationships in order to reveal distributional patterns. Particular taxa and groups of taxa (assemblages) of the sub-fossil diatoms are related to oceanographic parameters. Subsequently, environmental information retrieved from the assemblages shall help quantify paleoenvironmental and paleoclimatic reconstructions in the South China Sea.

## Materials & Methods

### Study area description

**Geomorphology:** The Beibu Gulf is a semi-closed fan-shaped continental shelf shallow sea in northwest SCS (Dong et al., 2019; Fig. 1). The water depth generally increased from northwest coastal areas towards the southeastward shelf break at about 200 m, but is less than 100 m in the middle and south of this gulf. However, water depth in broad areas in the northern part (north of 20°N) does not exceed 50 m. The middle Beibu Gulf has a relatively flat sea floor, while depths exceed 100 m in the southern part and near shelf break.

**Climate:** The research area has a strong monsoon climate. The winter is characterized by northeast monsoon, whereas during summer the southwest monsoon prevails with northward winds, and accompanying southeast wind. The northeast monsoon temporal interval is longer than the southwest one. The total annual rainfall is about 1,100–1,700 mm, and peak rainfall lasts from May to September (the rainy season) with an average monthly rainfall over 100 mm (Li et al., 2017).

**Temperature and Salinity:** Average monthly sea surface temperature of Beibu Gulf from 1994 to 2013 oscillated between 20.3–29.9 °C as derived from satellite data (Ya, Gao & Dong, 2015). In general, the spatial pattern of temperature distribution is cold water in the north and warm water in the south (Chen et al., 2009; Ya, Gao & Dong, 2015). The water temperature gradient between north and south usually amounts to ca. 5 °C, not exceeding 2 °C during the summer due to strong solar radiation over the entire SCS surface (Chen et al., 2009).

Due to higher fresh water runoff in the north and northwest, the northern Beibu Gulf has lower sea surface water salinities. On the contrary, the southern region is characterized by higher salinities that result from sea-water intrusion from SCS (Chen et al., 2009; Ya, Gao & Dong, 2015). The salinity displays seasonal variation with lower summer values due to fresh water discharge of ca. 21.2 psu in the coastal area. Winter salinity of coastal waters amounts to ca. 30.3 psu. In general, coastal area salinity ranges from 26 to 34 psu, but usually exceeds ca. 28 psu (Chen et al., 2009; Li & Li, 1993).

**Figure 2:**
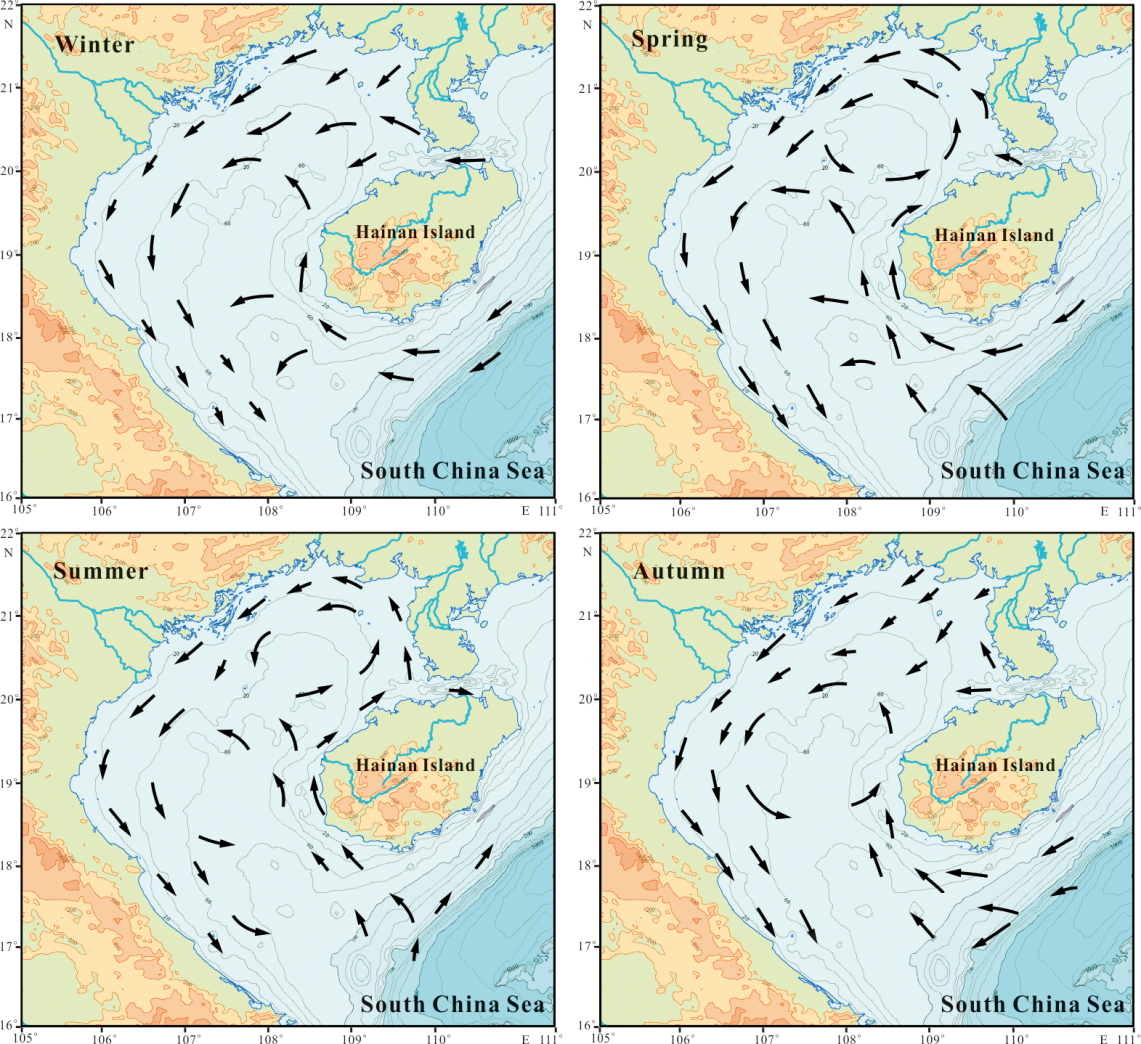
The circulation structures given in hydrography of China Seas (adapted from Su & Yuan, 2005).

**Water circulation:** Water circulation in Beibu Gulf is principally driven by wind speed and direction, sea-water density distribution, external open sea surface water mass and fresh water discharge, with the latter factor playing important role in long-term sea-water transportation (Wang et al., 2018). The northeast monsoon during winter induces a sinistral circulation, however, lengthy controversy continues over the summer circulation direction and mechanisms (Gao, Wu & Ya, 2017; Xia, Li & Shi, 2001). Summer circulation appears to follow a process where southeast monsoon induces a dextral circulation in Beibu Gulf, while the density gradient induces sinistral flow, with density currents being stronger than wind-driven, especially in the surface layer (Chen et al., 2009; Xia, Li & Shi, 2001; Su & Yuan, 2005). Also, westward flow through the Qiongzhou Strait plays an important role in formation of summer sinistral flow (Chen et al., 2019). In addition, coastal current flow from northern SCS along the southern Hainan Island coast plays an important role in Beibu Gulf circulation, particularly in winter to spring seasons (see Fig. 2).

### Laboratory measurement

Sixty-six surface box core sediment samples were collected from eastern Beibu Gulf (Fig. 1) during geological cruises organized by Guangzhou Marine Geological Survey, China during 2007 to 2012. We selected the core top 1st cm to analyze sub-fossil diatom assemblages, by permission from GMGS-CGS (No. GZH201500207).

**Diatom analysis:** Ten grams of dry sediment sample were processed at Xiamen University (China). Twenty ml of 30% hydrogen peroxide was used to oxidize organic matter, followed by 10 ml of 10% hydrochloric acid added to remove carbonates. Sieves (10 µm mesh size) were used to remove the large quantities of clay particles, and thereafter centrifuged twice with heavy ZnI_2_ liquid (density 2.4 g/cm^3^). Thirty µl of vigorously shaken cleaned diatom suspension was pipetted onto a 22 × 22 mm cover slip and left to dry at room temperature. The cover slips were transferred onto labeled slides, mounted with Naphrax and heated to remove air bubbles and humidity (Zhang et al., 2016). Three slides were prepared for each sample. At least 300 diatom valves were counted at the species or genus level in a slide made for each sample with a microscope magnification of ×400 and ×1000 (oil immersion).

Relative and absolute abundance of sub-fossil diatoms was estimated on the basis of diatom valve counts in particular slides. Diatom absolute abundance (=DAA) was calculated with the counted valves / weight of dry sediment used for sample processing. The diatom relative abundance (=DRA) was expressed as percentage of each diatom species/divided by total observed diatom abundance in each sample. The identification follows (Jin, Cheng & Lin, 1982; Jin, Cheng & Liu, 1992; Lan, Chen & Liu, 1995; Witkowski, Lange-Bertalot & Metzeltin, 2000; Guo & Qian, 2003). Separately, the freshwater diatom percentage, total percentage of warm water and higher salinity taxa, and planktonic to benthic (P/B) diatom ratio were calculated.

**Environmental data set:** The water depth of sampling site was recorded. The physical parameters of annual mean sea surface temperature (SST), annual mean sea surface salinity (SSS) and trophic state parameter of annual mean chlorophyll *a* concentration (Chl. *a*) data were abstracted through satellite remote sensing data set from the European Space Agency (European Space Agency, 2019). These four parameters were sorted as environmental variables in this study.

**Multivariate analysis:** In order to reduce the complex signal of sub-fossil assemblages, cluster analysis was used where diatom taxa with DRA in one individual site exceeded 5%. Statistically constrained cluster analysis of diatom species as groups was operated with PC-ORD version 5.0 (MjM Software Design). In this software, the Q-mode and R-mode hierarchical analyses are only based on diatom taxa in each site, the DRA as percentage was treated as a variable, utilizing Sorensen distance and Ward’s merging criterion.

Through correspondence analysis (CA) *via* the software Canoco 5.1 (Šmilauer & Lepš, 2014), the lengths of gradient (at 2.4 SD units) were obtained. The data were standardized in this software through log-transformation. Due to this gradient value, principal component analysis (PCA) was operated by unconstrained ordination suitably, in order to understand the relationship of diatom species matrix and environmental variables. The 2.4 SD units made redundancy analysis (RDA) valuable to reduce dimensions and obtain eigenvalue, following in constrained ordination model, for understanding the relationship of diatom species matrix and environmental variables.

## Results

### Diatom abundances

**Figure 3:**
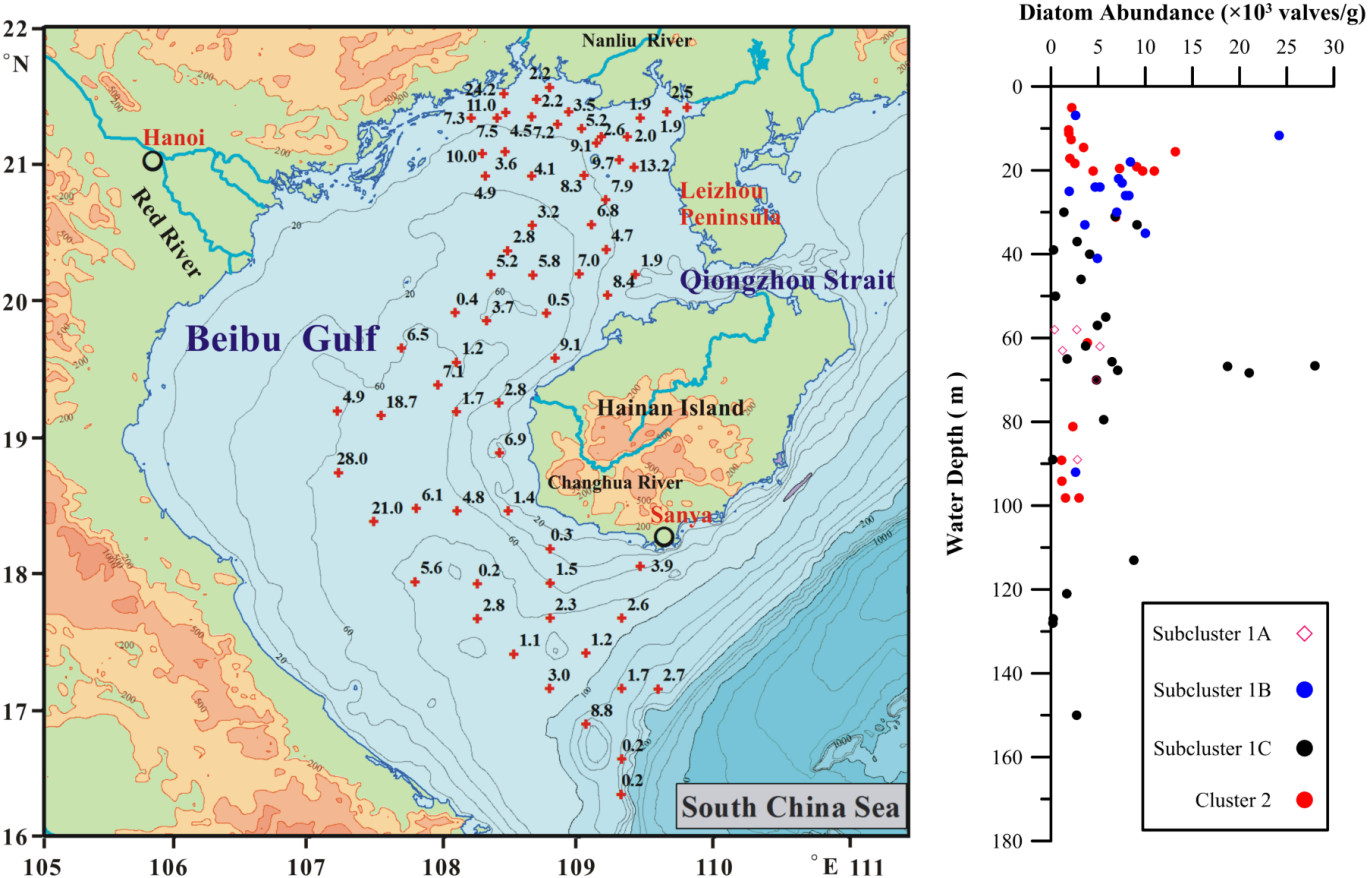
The diatom abundance in the Beibu Gulf topmost 1 cm. Left, spatial distribution; right, with water depth and showing the subclusters of sites listed in below text.

The DAA in studied sites varied within a broad range from 0.22 to 28.0 × 10^3^ valves/g, with average 5.53 × 10^3^ valves/g. DAA was higher in the middle and northern parts of inner Beibu Gulf in coastal area and Leizhou Peninsula (Fig. 3). The lower DAA values (below 1 ×10^3^ valves/g) were observed in two southernmost sites close to continental shelf break and four sites dispersed within middle Beibu Gulf.

### Diatom species

**Figure 4:**
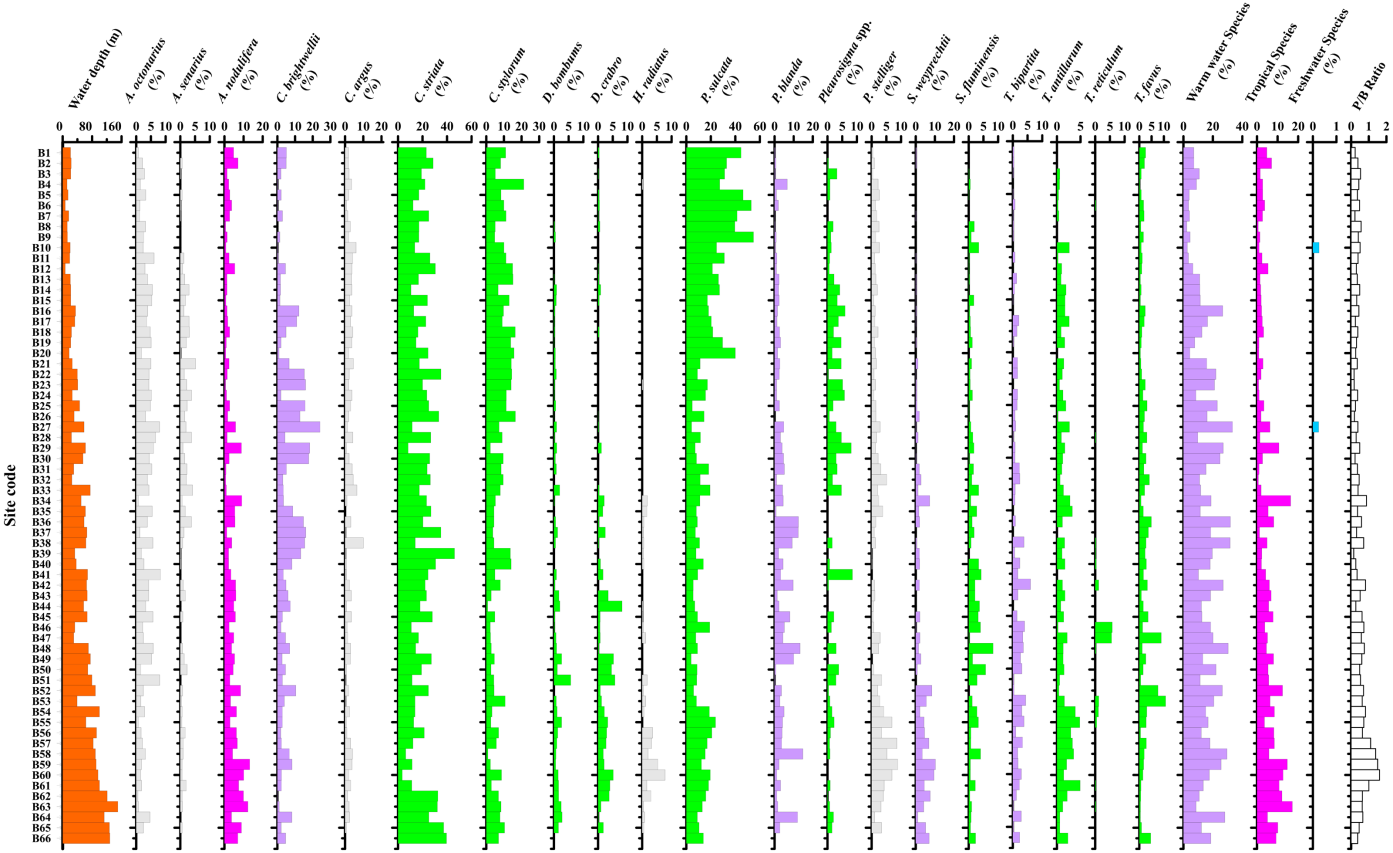
The dominant species distribution in the studied sites. The percentages of warm water, tropical, freshwater forms and planktonic to benthic diatom ratio is also illustrated.

**Figure 5:**
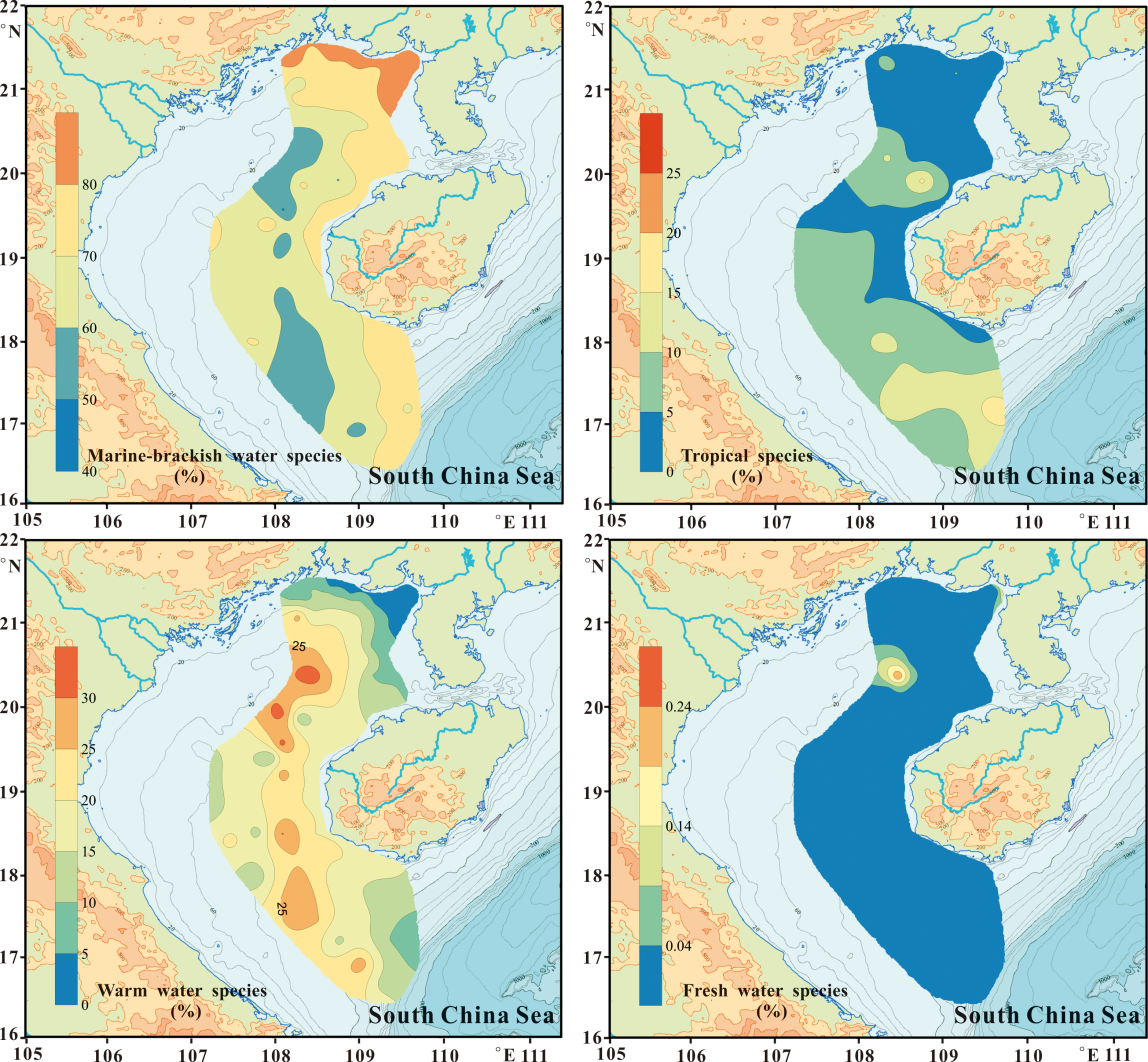
Diatom groups’ percentage of marine-brackish, tropical, warm water, and freshwater forms.

The surface sediment samples contained sub-fossil diatom assemblages usually represented by fairly high number of taxa. In total, 218 taxa (species) were identified with microscopic analysis based on above listed references. The identified diatoms with broad ecotypes include freshwater, marine-brackish water (coastal and neritic), warm water and tropical forms. The freshwater species *Planothidium hauckianum* and *Cymbella* sp. were identified in two sites (B10 and B27, Figs. 4, 5). Coastal benthic species in marine-brackish water forms widely occurred in this gulf and included *Cyclotella striata*, *C. stylorum*, *Diploneis crabro*, *Paralia sulcata*, *Trachyneis antillarum*, *T. aspera* and *Triceratium favus*.

A group of warm water species had wide distribution in the study area and included *Bacteriastrum hyalinum*, *Campylodiscus brightwellii*, *Ditylum brightwellii*, *Planktoniella blanda*, *Rhizosolenia bergonii*, *Stephanopyxis weyprechtii*, *Thalassiosira bipartita*, *T. leptopus*, and *Trieres chinensis*. The percentage of these warm water species ranged from 2.21% to 33.33% (average 15.52%, Fig. 4). The warm water group percentages were higher in middle and south Beibu Gulf, and similarly far away from the coastal zone (Fig. 5).

In the topmost sediment (highest 1 cm) also occurred numerous diatom species considered characteristic for tropical waters of higher salinity (*e.g.*, *Asterolampra vanheurckii*, *Asteromphalus flabellatus*, *Azpeitia africana*, *A. neocrenulata*, *A. nodulifera*, *Hemidiscus cuneiformis*). However, these species occurred with low DRA ranging from 0.63% to 17.15% (average 5.15%, Fig. 4). The tropical diatom forms achieved higher percentages in deep waters of southern and middle northern part of inner Beibu Gulf (Fig. 5).

**Figure 6:**
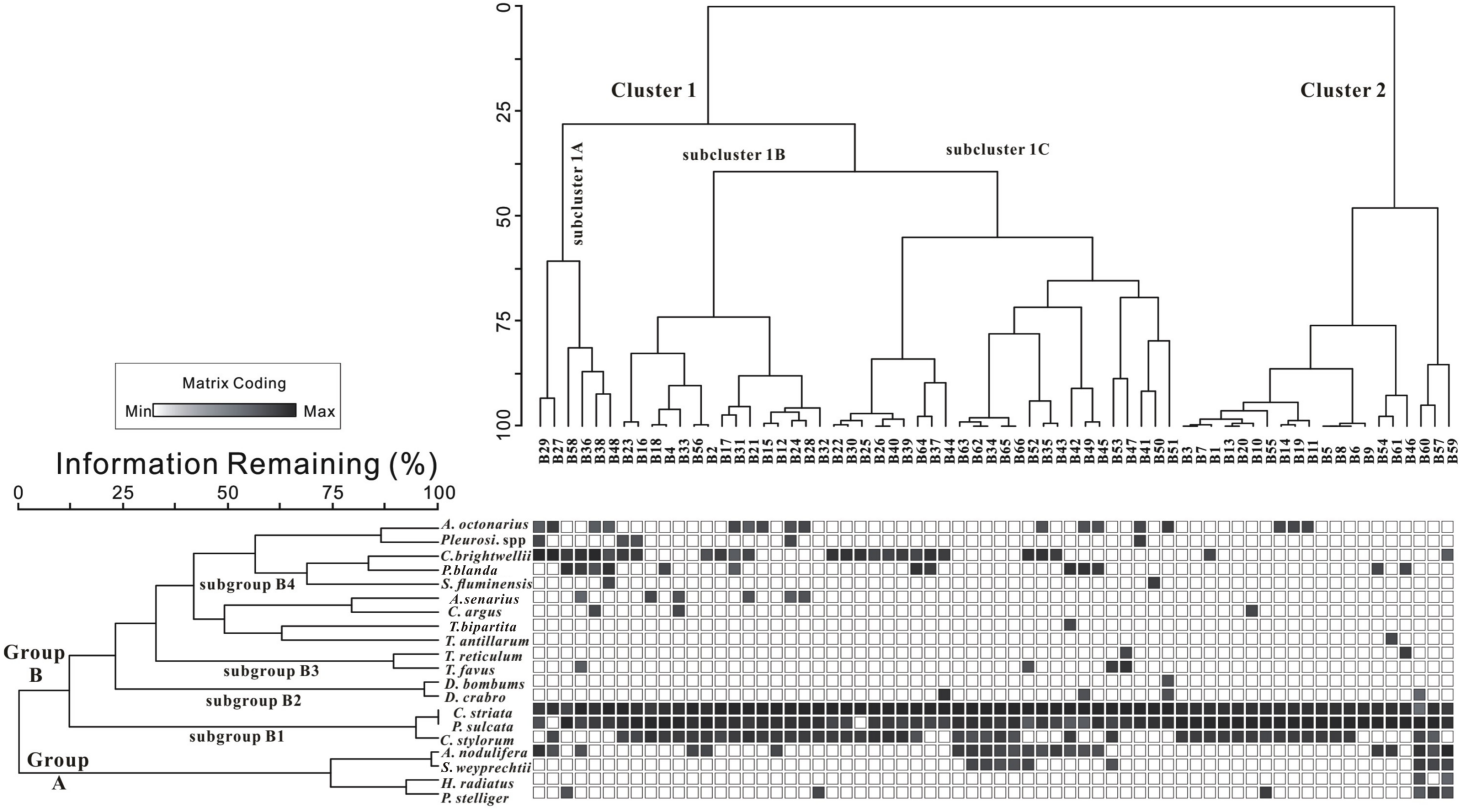
Q-mode cluster dendrogram (top) and R-mode cluster dendrogram (left). The R-mode dendrogram defines two groups (A and B) with four sub-groups (B1, B2, B3, and B4). The Q-mode dendrogram defines two clusters, the Cluster 1 and 2 with sub-clusters (1A, 1B, 1C).

The diatom P/B ratio ranged from 0.11 to 1.11 (average value 0.39). The diatom P/B ratio was below 1.0 in most sites, but showed a distinct increase in coastal areas, especially in shallow waters less than ca. 25 m water depth in northern Beibu Gulf. And increased P/B values occurred in middle Beibu Gulf over ca. 60 m water depth, and especially in southern Beibu Gulf where values exceeded 1.0 in three sites with deep waters. On the contrary, the P/B value show an amplitude function and without legible trend in areas where water depth between ca. 30 m to ca. 60 m (Figs. 4, 6).

Similarly to Pokras & Molfino (1986), we selected the diatoms species with relative abundance >5% in each sample which were considered as dominants. For the study area, 20 such species have been designated (Fig. 4, Table 1).

### Statistical analyses results

#### R-mode statistical analysis

The R-mode hierarchical cluster analysis is based on sites containing the 20 species with DRA exceeding 5% among all diatom taxa (Fig. 6). Two major groups were clearly distinguished, Groups A and B. Group B was further subdivided in subgroups B1, B2, B3 and B4.

Group A consisted of four species, *Azpeitia nodulifera*, *Stephanopyxis weyprechtii*, *Podosira stelligera* and *Hyalodiscus radiatus*, which on average amounted to 4.03%, 1.92%, 2.49% and 0.62%, respectively. *Azpeitia nodulifera*, a typical tropical species exhibited significantly higher relative abundances in southern part, and at a few sites in middle northern part of inner Beibu Gulf (Fig. 4). *Stephanopyxis weyprechtii* is a neritic warm water species, observed in this study with higher percentages in the southern part of this gulf. *Podosira stelligera* was commonly observed in southern Beibu Gulf in proximity to southwest Hainan Island. *Hyalodiscus radiatus*, a shallow water species, was rarely observed in this group but achieved higher relative percentages in the southern Beibu Gulf.

Group B1 consisted of three species, *Cyclotella striata*, *Paralia sulcata* and *Cyclotella stylorum*. These three species were considered typical coastal species and attained highest average percentages for the whole study area amounting to 21.17%, 17.97% and 8.11%, respectively (Fig. 4). *Cyclotella striata* showed higher relative abundances in the southernmost sites and in northern Beibu Gulf. Increased relative abundances of *P. sulcata* were observed in shallow waters of northernmost sites and percentages increased near southwest Hainan Island, in deeper waters (Fig. 4). *Cyclotella stylorum* showed higher relative percentages in the northeastern Beibu Gulf.

Group B2 included two species, *Diploneis bombus* and *D. crabro*. Both are coastal species, with low average DRA values amounting to 0.76% and 1.18%, respectively. The two taxa mainly occurred in middle Beibu Gulf.

Group B3 included two species, *Triceratium favus* and *T. reticulum*. Both are coastal species, with lower average DRA value about 2.09%, 0.26%, respectively. *Triceratium favus* is widely distributed in studied area and with higher relative abundances in sites near southwestern Hainan Island and a few sites of middle Beibu Gulf. *Triceratium reticulum* was less abundant in similar sites, likewise with increased relative abundances along southwestern Hainan Island coasts.

Group B4 consisted of nine species that were relatively abundant, but randomly distributed. *Actinocyclus octonarius*, *Campylodiscus brightwellii*, *Coscinodiscus argus* and *Planktoniella blanda* attained average DRA values exceeding 2.5%, whereas remaining species were below 1.8%. *Campylodiscus brightwellii* was observed with higher relative percentages in middle northern Beibu Gulf and in a few sites in southern Beibu Gulf. The average DRA values amounted to 5.94%. *Actinocyclus octonarius* quite commonly achieved average DRA of 3.47%, except in the southernmost site B66. Increased relative percentages of this species were observed in northern Beibu Gulf, whereas lower values occurred in southern Beibu Gulf. Except for site B66, *P. blanda* belonged to a group of dominant species in the studied area with average DRA of 3.57%. Enhanced relative percentages were observed in middle Beibu Gulf. *Coscinodiscus argus* commonly occurred in the studied area, however, did not achieve increased percentage (ca. 2.5%) with highest DRA observed in the northern part of Beibu Gulf.

#### Q-mode statistical analysis

To analyze the relationships among these studied sites, a Q-mode hierarchical cluster analysis was conducted (Fig. 6). The resulting dendrogram represents a grouping of sites according to species’ DRA, and two major clusters, Clusters 1 and 2 are recognized. Cluster 1 can be further subdivided into three subclusters, 1A, 1B and 1C (Fig. 6). In order to clearly express these dominant diatom species in the Q-mode hierarchical cluster, this article sorts diatom species’ average DRA over 10% in one sub-cluster as major dominant diatom species, and species with average DRA less than 10% are chosen as minor dominant diatoms.

**Figure 7:**
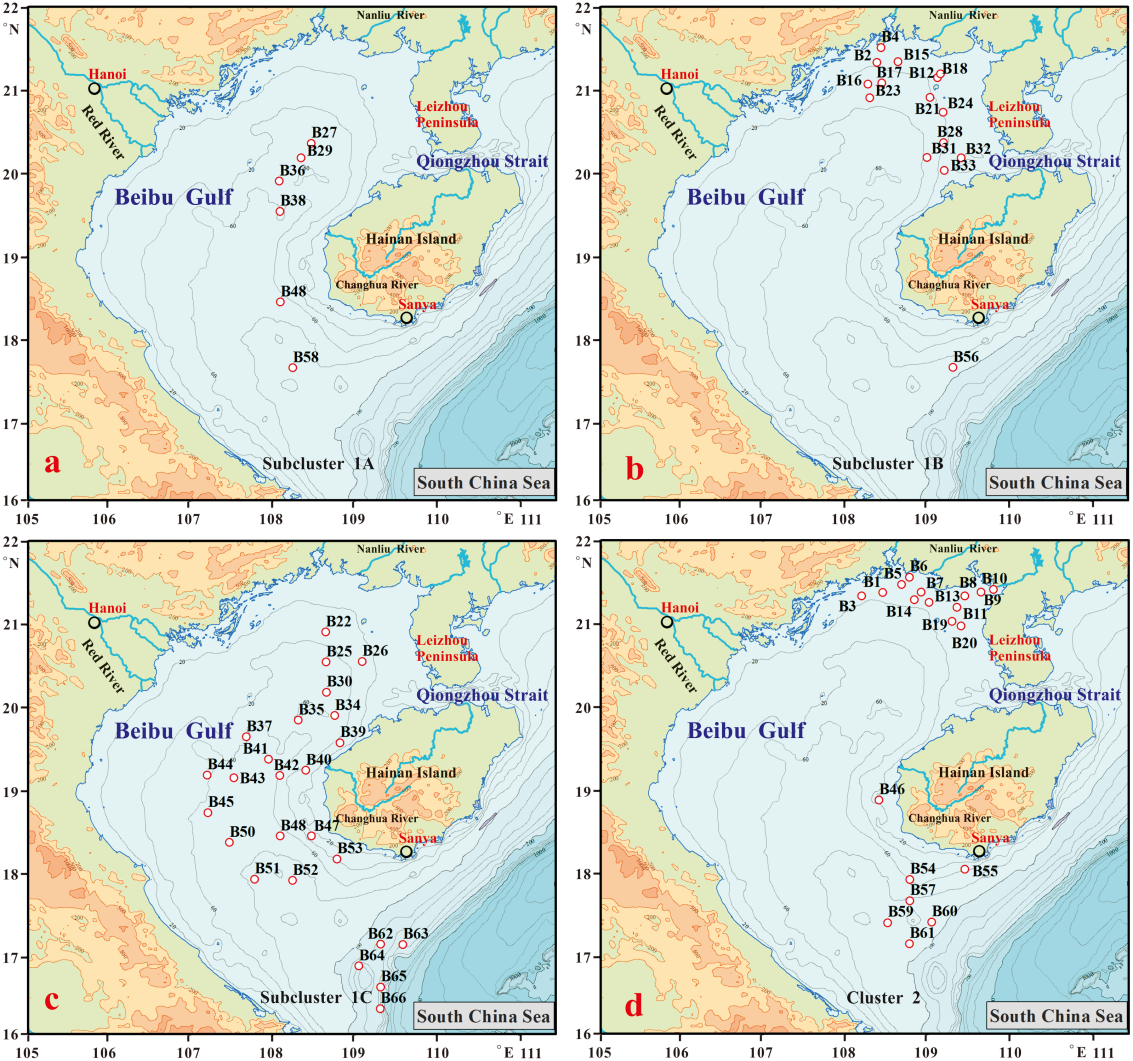
Distribution of the sites studied within the four subclusters 1(A), 1(B), 1(C) and Cluster 2(D).

Subcluster 1A comprised six sites, which are in middle Beibu Gulf located from north to south with water depths that exceed 50 m (Fig. 7), and mainly dominated by *Campylodiscus brightwellii* and *Cyclotella striata*, with increased percentages of *Paralia sulcata* and *Planktoniella blanda* (Table 2)*.* However, three other diatom species occurred with lower abundances, *Actinocyclus octonarius*, *Azpeitia nodulifera*, *Coscinodiscus argus* in this subcluster.

**Table 2:** The most abundant diatom species in the four subclusters. The species are ranked according to decreasing average DRA over 1.5% in each subcluster.

Subcluster	Dominant diatom species	
	Major dominant species (average DRA < 10%)	Minor dominant species (average DRA < 10%)
Subcluster 1A		*Paralia sulcata* (8.59%)
	*Campylodiscus brightwellii* (14.39%)	*Planktoniella blanda* (8.30%)
	*Cyclotella striata* (12.49%)	*Actinocyclus octonarius* (4.20%)
		*Azpeitia nodulifera* (3.35%)
		*Coscinodiscus argus* (1.67%)
Subcluster 1B	*Cyclotella striata* (22.23%)	*Campylodiscus brightwellii* (3.72%)
	*Paralia sulcata* (18.96%)	*Actinocyclus octonarius* (1.83%)
	*Cyclotella stylorum* (11.60%)	
Subcluster 1C	*Cyclotella striata* (27.06%)	*Paralia sulcata* (8.90%)
		*Cyclotella stylorum* (5.75%)
		*Campylodiscus brightwellii* (5.57%)
		*Azpeitia nodulifera* (3.35%)
		*Thalassiosira bipartita* (2.09%)
		*Stephanopyxis weyprechtii* (1.64%)
Cluster 2	*Paralia sulcata* (30.93%)	*Cyclotella stylorum* (7.00%)
	*Cyclotella striata* (15.62%)	*Azpeitia nodulifera* (2.19%)
	*Cyclotella stylorum* (7.00%)	*Podosira stelligera* (1.56%)

Subcluster 1B comprised fifteen sites, mainly located in northern Beibu Gulf, with only one site (B56) located southwest of Hainan Island near Sanya City (Fig. 7). In this subcluster, the major dominant diatom species were *C. striata*, *Cyclotella stylorum* and *P. sulcata*, with *Campylodiscus brightwellii* and *A. octonarius* as minor dominant diatom species.

Subcluster 1C comprised twenty-five sites occur mainly in middle and southernmost Beibu Gulf and along the west coast of Hainan Island (Fig. 7). *Cyclotella striata* is the only major dominant species in this subcluster, but other species (*e.g.*, *C. brightwellii*, *C. stylorum*, *P. sulcata* and *Thalassiosira bipartita*) with higher average DRA graded as minor dominant species. Thus, this subcluster gathered various dominant diatom species, which suggests these to be in a strongly mixed area.

Cluster 2 comprised twenty sites, mainly located in northernmost Beibu Gulf, west of Leizhou Peninsula, and southwestern coast near Sanya City (Fig. 7). *P. sulcata* and *C. striata* were major dominant species in this cluster, except for site B59 with *A. nodulifera* over DRA 13% as most dominant species. In this cluster, *C. stylorum* also has relatively higher percentages, usually over 5%.

#### PCA and RDA ordination analysis

**Figure 8:**
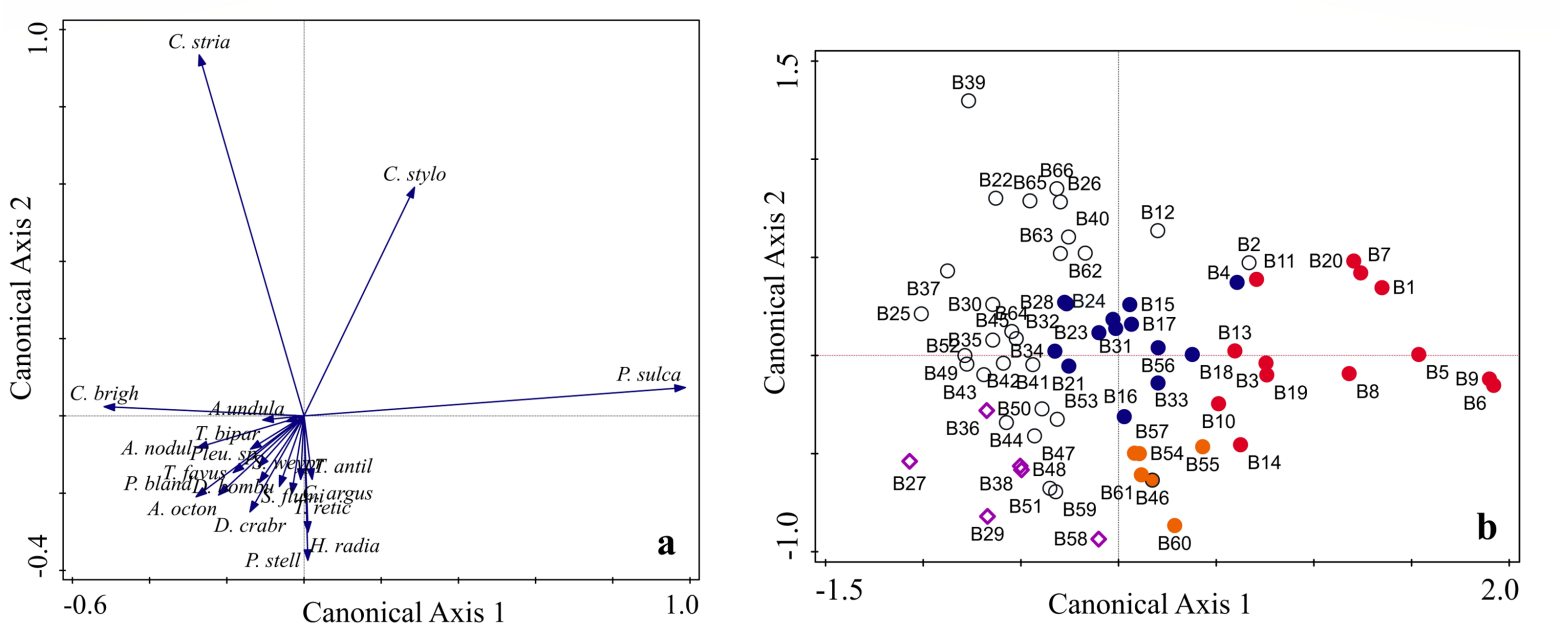
PCA analysis of diatom species within the sties studied ((A) is species plot; (B) is the sites ordination). (B) Diamonds show the site in subcluster 1A. Green circles show the site in subcluster 1B. Blank circles show site in subcluster 1C. Red circles show the site in Cluster 2 in northeastern Beibu Gulf. Orange circles show site in Cluster 2 in south Beibu Gulf.

The PCA analysis of diatom species showed Axis 1 to Axis 4 with eigenvalues 0.4667, 0.2112, 0.0968, 0.0677, respectively (see Table 3). This explained 84.24% of (cumulative) variation. The PCA analysis results can make clearly the ordination of dominant diatom species in the distribution pattern. *C yclotella striata*, *C. stylorum* and *Paralia sulcata* were the most significant species as illustrated in Fig. 8A, while other diatom species were of lower weight in the matrix. The studied site plot in the PCA analysis (Fig. 8B) showed an order distribution that corresponds to the results of Q-mode hierarchical cluster analysis.

**Table 3:** The PCA analysis results for the first four axes.

		Axis 1	Axis 2	Axis 3	Axis 4
Analysis type	Unconstrained				
Method	PCA				
Total variation	23672.702				
Statistic Eigenvalues		0.4667	0.2112	0.0968	0.0677
Explained variation (cumulative)		46.67	67.79	77.47	84.24

Subcluster 1A gathers in corner of the third quadrant, while subcluster 1B gathers in center of the graph (Fig. 8B). Subcluster 1C mainly gathers in the fourth quadrant. Cluster 2 crossed the first and second quadrant. Sites in Cluster 2 along the PCA coordinate axes (Fig. 8B) with higher abundance of *P. sulcata*.

**Figure 9:**
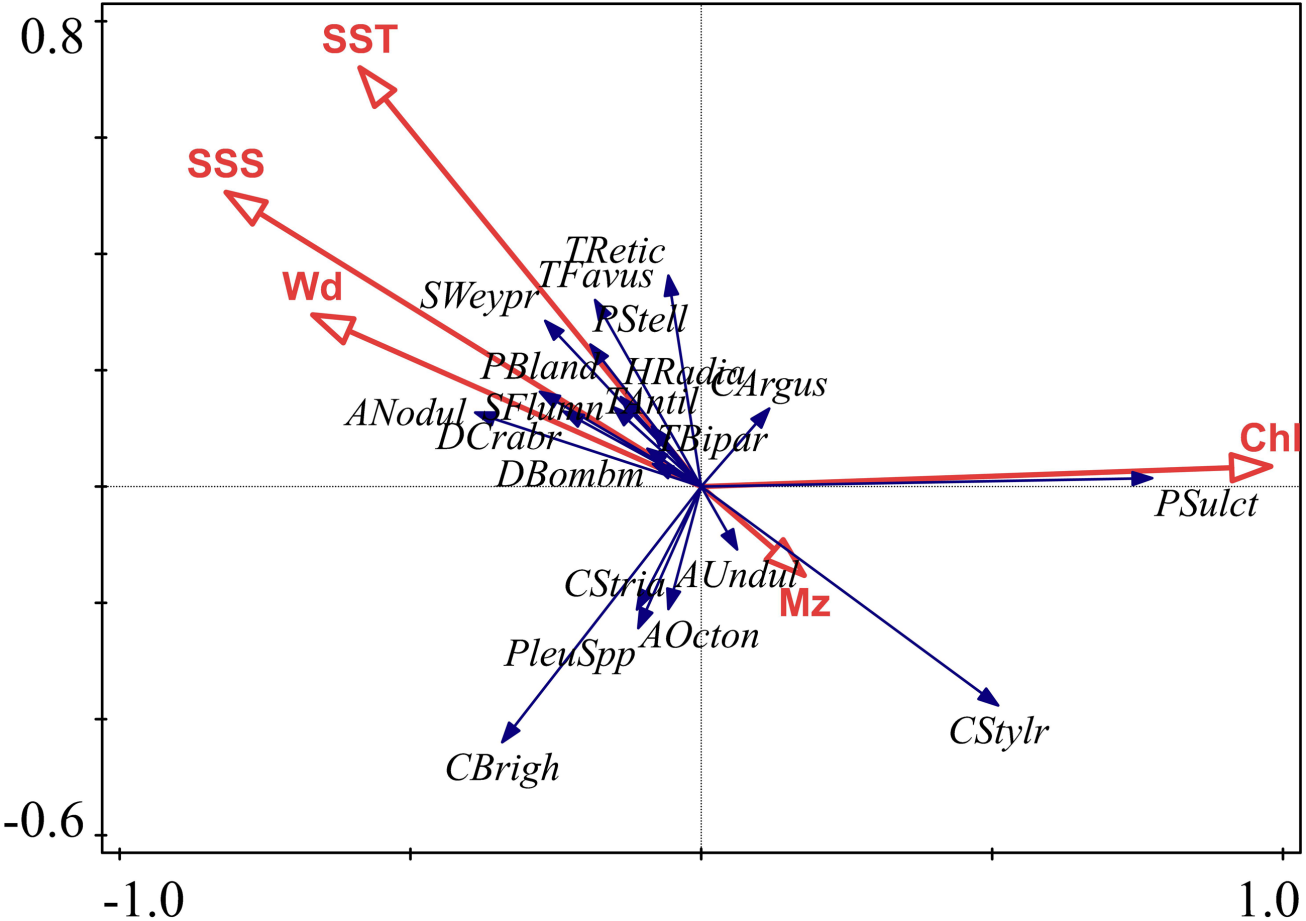
Plot of diatom species and environmental variables by RDA analysis.

The RDA analysis between diatom species and environmental variables showed these four explanatory variables to account for 38.14% of variation (Table 4, Fig. 9). The statistic axes 1 to 4 have eigenvalues of 0.2985, 0.0507, 0.0229, 0.0093, respectively (see Table 4). These correspond to the species distribution (*A. nodulifera*, *P. blanda*, *S. weyprechtii*), attributed to deeper-water, higher temperature and salinity sites in middle and south Beibu Gulf. The Chl. *a* mainly correspond to *C. striata* and *P. sulcata* (two top species of higher average abundances in this study, Table 1) which occur mostly in north and southeastern Beibu Gulf.

**Table 4:** The RDA analysis results for the first four axes.

		Axis 1	Axis 2	Axis 3	Axis 4
Analysis type	Constrained-P				
Method	RDA				
Total variation	23672.702				
explanatory variables account	38.14%				
Statistic Eigenvalues		0.2985	0.0507	0.0229	0.0093
Explained variation (cumulative)		29.85	34.92	37.2	38.14
Pseudo-canonical correlation		0.8131	0.5935	0.47	0.3595
Explained fitted variation (cumulative)		78.27	91.56	97.56	100
Permutation Test Results	pseudo-F = 9.4, *P* = 0.002				

## Discussion

Various environmental factors affect sub-fossil diatom distribution, including water depth, temperature, salinity, substrate type, and nutrient levels driven by seasonal surface flow and water circulations. Even at sites with similar environmental parameters, the species composition of diatom assemblages and percentages of dominant taxa vary significantly. This observation suggests that environmental proxies and diatom assemblages as clusters are related in a rather complicated way.

### Cluster’s attribution explanation

On the clusters level, Cluster 1 and Cluster 2 occupied about 2/3 and 1/3 of sites in the studied area, respectively. This ratio indicates that major factors largely impact the shallow gulf, although the two clusters are dominated by different diatom species and ordination linked with DRA.

### Subcluster 1A formation

Distribution of subcluster 1A is distinctness to align in middle Beibu Gulf from ca. 50 to 90 m water depth. The main dominant species is the marine benthic diatom *C. brightwellii*, associated with warm water (Jin, Cheng & Lin, 1982; Jin, Cheng & Liu, 1992), and *A. nodulifera* as minor dominant species indicative of open sea higher temperature and salinity (Winter, 2001). While, the increased relative abundance of *C. striata*, *C. stylorum* and *P. sulcata* coastal species as the main PCA plotted variations (Witkowski, Lange-Bertalot & Metzeltin, 2000; Guo & Qian, 2003; Zong, 1997) indicates impact of coastal currents in middle Beibu Gulf (see Figs. 3, 4 and 8).

### Subcluster 1B formation

Subcluster 1B is observed in northern Beibu Gulf with water depth decreasing to ca. 40 m, except for one site (B56) at ca. 90 m water depth in south Beibu Gulf. Compared to subcluster 1A, 1C and Cluster 2, this subcluster is likely to occur in a transition area, bounded to south by subcluster 1A and 1C, and northward linked to Cluster 2. This directly explains formation related to oceanographic conditions in terms of coastal water impact and currents driven from Qiongzhou Strait (Fig. 2), mixing with warm water from SCS. The dominant species *C. striata*, *C. stylorum* and *P. sulcata* occupied most part of this area, as typical coastal species, that implied coastal environment importance in subcluster origin. Common occurrence of *C. brightwellii* (associated with warm conditions) and *A. octonarius* (marine planktonic species, Witkowski, Lange-Bertalot & Metzeltin, 2000; Guo & Qian, 2003) in this shallower area shown as minor dominant species in Table 2, provides an effective proof that this area was impacted by other water masses.

### Subcluster 1C formation

Subcluster 1C included the largest set of sampling sites in Beibu Gulf with large spatial distribution but divided into two areas (Fig. 7). Comparing to Subcluster 1B, sites positioned in middle Beibu Gulf have a mixed regime with diverse diatom species in terms of their requirements. The typical coastal species *C. striata* is the only major dominant species in this subcluster to indicate strong impact of coastal waters, but there are other species with average DRA ranging from 5% to 10% (Table 2). Warmer water and higher salinity species occurred, including *A. nodulifera*, *S. weyprechtii* and *T. bipartita*, which indicate warm water from open SCS invading into middle and north Beibu Gulf.

An interesting observation has been made regarding the highest DAA in this area (Fig. 3) which occurred in middle Beibu Gulf (site B45). This station is located within the Red River discharge plume, which can be source to increased diatom discharge and deposition.

In the southernmost part of studied area (sites B66 and B65) near shelf break (Fig. 1), the lowest DAA (Fig. 3) was collaborative with dominance of coastal species and *C. striata* in particular. Although increased temperature and salinity conditions proximity of the open SCS water mass, these stations would not rather display higher DRA of tropical water species. The explanation can be either a strong downslope current that results from a high sediment surface gradient or occurrence of reworked sediments.

### Cluster 2 formation

Cluster 2 appeared in two relatively independent areas in the northernmost and south parts of Beibu Gulf (Fig. 7). The highest average DRA of *Paralia sulcata* differentiates this cluster from above subclustered sites. The northernmost Beibu Gulf received siliceous acid by runoff (Wang et al., 2015), it provides optimum conditions for brackish water diatom growth and form large depositional flux in these cluster sites (Table 2). In line with this interpretation is the presence of freshwater forms which have been observed at B10 and B27 in the northern coastal area. Meanwhile, north SCS coastal currents transferred coastal water into Beibu Gulf *via* the Qiongzhou Strait, which increased presence of brackish/coastal species (*e.g.*, *C. striata* and *P. sulcata*).

*Azpeitia nodulifera* is the most dominant species in one site (B59), in southern Beibu Gulf, which suggests strong impact by warm and high salinity water from SCS. While *P. sulcata* and *C. striata* were abundant (Table 2) in a few sites in south Beibu Gulf, these were likely transported from inner gulf by the coastal current southeastern of Hainan Island.

### Role of critical environmental factors

#### Nutrients

Due to lack of *in-situ* measurement and nutrient data, the Chl. *a* is applied as a proxy corresponding to trophic state. The nutrient conditions support diatom growth in this study area (Zhou et al., 2008). The Chl. *a* not only show the longest vector but also highlight the significance of *P. sulcata* (Fig. 9). In the shallow water area, nutrients are available for brackish-water species which seem to be competitive in terms of nutrient uptake to promote growth, and one such species is likely *P. sulcata*. Silicon, mainly in the form of siliceous acid discharged from riverine runoff (Witon et al., 2006) fostered the abundance of heavily silicified diatoms (*e.g.*, *Paralia sulcata*, McQuoid & Nordberg, 2003) that indicate that the estuary and coastal zones in studied area provided optimum conditions for brackish water diatom species. In generally, the Chl. *a* value has a weak liner trench against diatom abundance on these sites. While, upon on too much more factors impacted on complicated diatom abundance distribution as Fig. 3 shown, the sites have low value Chl. *a* with higher diatom abundances, vice versa, the opposite is also happened.

#### Salinity

The SSS is variable in the second axis (Fig. 9) to influence diatom distribution in this area. The brackish water species were dispersed widely over the entire area and at some stations even dominated the sub-fossil assemblages. Included in this group were *A. octonarius*, *C. striata*, *C. stylorum* and *Podosira stelligera*. This is an interesting phenomenon since sea-water annual salinity of Beibu Gulf is relatively high and exceeds 28 psu, except for lower values near the coast and estuary (Chen et al., 2009). The abundance of brackish water diatoms is interpreted to indicate strong river runoff that supports growth of brackish water species that were laterally transported to provide the high abundance of strongly silicified taxa. Relative abundances of *P. sulcata* over 90% have been reported by Zong (1997) from Scottish coastal lakes, Witkowski et al. (2005) from the Western Baltic Sea, and Witon et al. (2006) from Faroe Island neritic zone. However, in this study strongly silicified *Cyclotella* taxa (*e.g.*, *C. striata* and *C. stylorum*) were also subjected to lateral transport and concentrated by lateral flow in Beibu Gulf.

#### Temperature

Although the water temperature gradient is not large in Beibu Gulf, Fig. 9 shows SST to be variable in the second axis and influence diatom distribution in this area. In the sites we studied, percentages of warm and tropical species are shown in Figs. 5 & 6 Higher values occur in southern Beibu Gulf; in site B59 the typical tropical *A. nodulifera* (*e.g.*, Guo & Qian, 2003) was observed to be a dominant species. This observation strongly supports warmer water inflow into the gulf from the southward situated South China Sea basin. The presence of tropical water indicative diatom taxa extends to middle Beibu Gulf, which may be considered the limit of open sea warmer water penetration into the area.

#### Water depth

Although a subordinate environmental variable when compared to SSS, SST and Chl. *a* in this study area (Fig. 9), water depth appears to be one of the critical factors that controlled sub-fossil diatom distribution. As revealed by cluster analyses (Figs. 3, 6, 7), the subcluster 1B sites were grouped in northern Beibu Gulf in relatively shallow water (below 50 m). Subcluster 1A is arranged along the ca. 50 to 90 m water depth contours. In Cluster 2, the sites mainly originate in the northern coastal area with water depths below 25 m. Dominant in all of these sites, but especially in northern shallow water areas is *P. sulcata*, a typical coastal species. This observation differs from Jiang (1987), who commented that sub-fossil *P. sulcata* mainly followed the water depth increase to enhance relative abundance on the East China Sea shelf. While, in the western Taiwan Strait, this species has higher abundance in surface sediments of less than 50 m water depth (Zhang, 2009). This may show *P. sulcata* to be a regional specialist species with preferred water depth that varies with region. In addition, *P. sulcata* dominated a few sites of Cluster 2 located in southern Beibu Gulf deep water. This is probably related to other factors than water depth, such as trophic state (as revealed by Chl. *a* in Fig. 9) and lateral current transports as discussed in above context.

In middle Beibu Gulf, especially in areas where water depth exceeds 25 m, diatom assemblage is composed of planktonic forms (cf. subcluster 1C shown in Fig. 7) typical for sedimentation in calm conditions. However, that this assemblage accommodates diatoms from various types of habitats (benthic and planktonic) may imply influence by middle Beibu Gulf currents that transported diatoms to ca. 90 m depths (Fig. 2). The steeper bottom gradient with coarse sediments substrate type, in the southernmost part of this study area to over ca. 130 m depths close to shelf break (Fig. 1), created the anomalous mixture of diatom assemblage in subcluster 1C.

**Figure 10:**
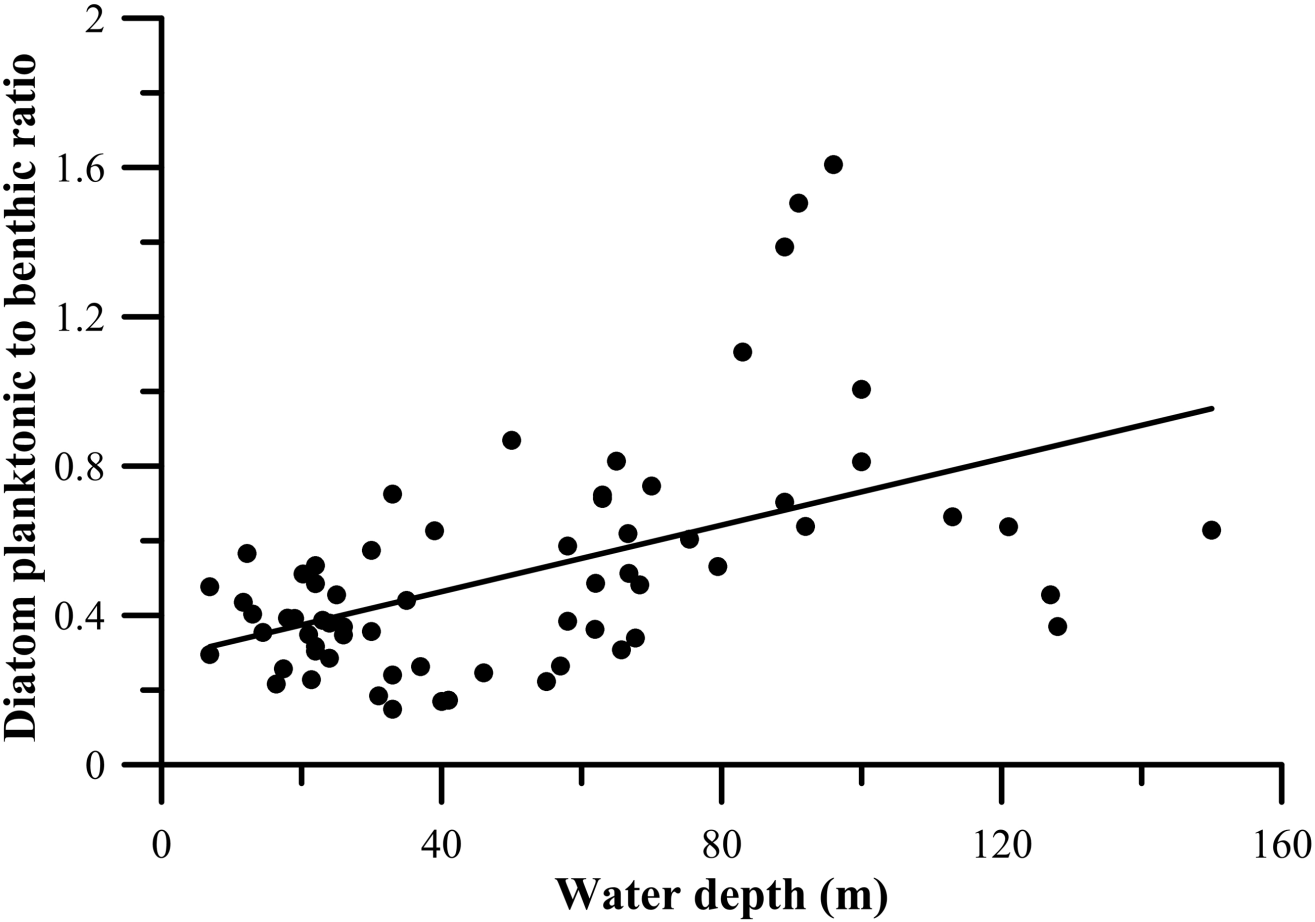
Diatom planktonic to benthic ratio (P/B ratio) against the water depth.

The higher diatoms P/B ratio were mainly observed in southern Beibu Gulf, and also occur in the middle area with deeper water (Figs. 4, 10). The lower ratio may be limited mainly to coastal areas with lower water depths. This P/B ratio is directly related to water depth in the studied area. However, differences from this general pattern were observed in the southernmost sites B65 and B66 with relatively deep water, which revealed lower P/B ratio due to abundant occurrence of coastal species. Usually, the planktonic diatoms inhabit water masses with higher depths, whereas benthic diatoms mainly grow in shallow water photic conditions. Numerous case studies have used the P/B ratio to qualitatively infer water-level changes in lakes (*e.g.*, in Africa, Ekblom & Stabell, 2008; in Asia, Wang et al., 2013), while in the marine system literature this index is less popular. This study may expose water depth constraining diatoms P/B ratio in a broad sense.

### Statistical significance for modern and paleoenvironment understanding

Forward the previous quality level studies on sub-fossil diatom in Beibu Gulf (Wang, Jiang & Zhang, 1990; Zhang, Chen & Zhang, 2013), this statistical analysis provides clear information about the sub-fossil diatom distribution pattern in this gulf, which has a complicated environment in terms of oceanographic proxies and hundreds of diatom taxa. The nutrients, seawater salinity, temperature, water depth, water mass currents, and river runoff variables may deeply impact the diatom distribution in surface sediments from the studied area. The Chl. *a*, SST, SSS and water depth, explain 38.14% of environmental information to impact the diatom species distribution in this gulf. This indicates that other locally significance parameters also impacted the sub-fossil diatom species distribution in this study area.

Except for seawater salinity and temperature constraints on diatom distribution among the subclusters, discussion in connection with water depth indicates that different subclusters have significance for modern environment management based on statistical analysis and understanding. In particular, the ca. 25 m water depth is a lower boundary to northernmost sites in Cluster 2, creating a relative biogeographical area, of potential value for regional environmental protection management in inner Beibu Gulf. This result provides a potential candidate/selection index for a marine ecological red-line definition for sustainable development, hence can be of some help to marine and coastal zone management.

The relationship is perspicuity in qualitative description *via* this statistical analysis, which contributes to a useful direction for further paleoceanographic and paleoenvironmental study in this region. Furthermore, given combined measurement oceanographic parameters, the quantitative analysis is valuable for statistical relation interpretation to sub-fossil distribution in Beibu Gulf.

## Conclusions

This study of sub-fossil diatoms preserved in Beibu Gulf surface sediments explored the biotic distribution pattern and for the first time involved a relatively large area. The diatom abundance, species and analysed clusters are related to the oceanographic proxies, which provides a useful basis for further paleoceanography and paleoenvironment work in this region. (1) Coastal diatom species are widely distributed in all sites in this gulf. In particular, this concerns *Cyclotella striata*, *Paralia sulcata* and *Cyclotella stylorum*, all characterized by relatively high abundances. Freshwater diatoms were observed in the northern Beibu Gulf. Warm water diatom species mainly appeared in the southern Beibu Gulf, which matched the diatom planktonic to benthic ratio distribution. The R-mode cluster analysis of sub-fossil diatom species split these into Groups A and B (with four sub-groups B1, B2, B3 and B4) and showed quite a complex diatom species distribution pattern. The Q-mode cluster analysis has shown clear site distribution of sub-fossil diatoms divided into various areas. (2) The available environmental variables include annual mean sea surface temperature, annual mean sea surface salinity, mean sea-water chlorophyll *a* concentration, and water depth, as shown with the canonical redundancy analysis (RDA), constrain diatom species distribution, although these explain only 38.14% of variation. Trophic state represented by chlorophyll *a* concentration is the major ordination factor to impact diatom distribution. (3) Although water depth is a subordinate factor compared to the above three variables determined by RDA analysis, it controls the sub-fossil diatom distribution in the studied area. In waters below 25 m depth, *Paralia sulcata* was dominant and this assemblage is limited to the northernmost Beibu Gulf. The diatom planktonic to benthic ratio tends to respond to deep water in this gulf. (4) The significant variation in sub-fossil diatom species abundance and distribution in studied area indicates that apart from nutrient availability and water depth, sea temperature, salinity and other factors such as regional river runoff and currents jointly influence the diatom assemblages. The regional water circulation seems to play an important role as external driver in transporting the open sea warmer and higher salinity masses into the south and middle part of this gulf, which corresponds with higher percentages of tropical diatom forms. On the other hand, the coastal currents from north of South China Sea flow into northern Beibu Gulf through the Qiongzhou Strait and to the southern part of this gulf along south Hainan Island coasts, which is marked by higher percentages of *Paralia sulcata* and *Cyclotella striata*.

##  Supplemental Information

10.7717/peerj.13115/supp-1 Supplemental Information 1 Raw Data

## Additional Information and Declarations

### Field Study Permissions

The following information was supplied relating to field study approvals (i.e., approving body and any reference numbers): Guangzhou Marine Geological Survey, China Geological Survey, China.

### Data Availability

The following information was supplied regarding data availability: The diatom identification data used in this article is available in the Supplemental Information. Other resarchers may use or re-use it to do other statistical analysis.
